# Disrupted functional connectivity of periaqueductal gray subregions in episodic migraine

**DOI:** 10.1186/s10194-017-0747-9

**Published:** 2017-03-21

**Authors:** Zhiye Chen, Xiaoyan Chen, Mengqi Liu, Shuangfeng Liu, Lin Ma, Shengyuan Yu

**Affiliations:** 1 Department of Radiology, Chinese PLA General Hospital, 28 Fuxing Road, Beijing, 100853 China; 2 Department of Neurology, Chinese PLA General Hospital, 28 Fuxing Road, Beijing, 100853 China; 3 Department of Radiology, Hainan Branch of Chinese PLA General Hospital, Sanya, 572013 China

**Keywords:** Episodic migraine, Functional connectivity, Magnetic resonance imaging, Periaqueductal gray, Resting-state functional MRI

## Abstract

**Background:**

The periaqueductal gray (PAG) dysfunction was recognized in migraine, and the altered dysfunction of PAG subregions were not totally detected up to now. The aim of this study is to investigate the altered functional connectivity of PAG subregions in EM patients.

**Methods:**

The brain structural images and resting state functional MR imaging (rs-fMRI) data were obtained from 18 normal controls (NC) and 18 EM patients on 3.0 T MR system. Seven subregions of PAG were classified as bilateral ventrolateral PAG (vlPAG), lateral PAG (lPAG), dorsolateral PAG (dlPAG) and dorsomedial PAG (dmPAG). The functional connectivity maps of each PAG subregion were calculated, and Two sample *t*-test was applied with age and sex as covariables.

**Results:**

Bilateral vlPAG and left dlPAG presented decreased functional connectivity, and the other subregions (bilateral lPAGs, right dlPAG and dmPAG) showed no significant altered functional connectivity in EM compared with NC. The brain regions with decreased functional connectivity mainly located in bilateral prefrontal cortex(PFC), middle temporal gyrus, primary motor area (PMA) and supplementary motor area (SMA) and right ventrolateral PFC (vlPFC) in EM patients in this study. Disease duration was positively related to the functional connectivity of bilateral vlPAG on the bilateral thalamus and putamen, left pallidum and right medial orbitofrontal gyrus in EM patients.

**Conclusion:**

The present study suggested that the dysfunction of bilateral vlPAG and left dlPAG presented in EM, and functional evaluation of PAG subregions may be help for the diagnosis and understanding of EM pathogenesis.

## Background

Periaqueductal gray (PAG) was a center with powerful descending antinociceptive neuronal network [1, 2], and the dysfunction in migraine [3] may be considered as a possible “generator” of migraine attacks [1, 4, 5]. Current studies [6, 7] supported that PAG network participated in the migraine, which may impair the descending pain modulatory. In episodic migraine (EM) paitents, it had been detected for the nonspecific PAG lesions on T2WI [8], impaired iron homeostasis of PAG [1], and increased mean kurtosis and mean diffusivity on diffusion kurtosis imaging [9].

PAG is a small mesencephalic brain structure, and the different subregions may present different specific function. Existing concepts recognized that the anatomical organization of PAG mainly included seven parts: bilateral ventrolateral PAG (vlPAG), bilateral lateral PAG (lPAG), bilateral dorsolateral PAG (dlPAG) and dorsomedial PAG (dmPAG), and PAG connections could also be identified by diffusion tractography and functional connectivity in mammals [10].

The previous document [11] demonstrated that the vlPAG was functionally connected to brain regions associated with descending pain modulation (anterior cingulate cortex (ACC), upper pons/medulla), lPAG and dlPAG were connected to brain regions with executive functions (prefrontal cortex, striatum, hippocampus). However, the precise neuromechanism of PAG subregions in the descending pain modulatory system [7] in migraine is not clear.

To investigate the descending pain modulatory circuitry of PAG subregions in migraine, we hypothesize that PAG subregions present different functional connectivity patterns in migraine. To address this hypothesis, we prospectively obtained high resolution structural images and resting state functional MR imaging (rs-fMRI) from 18 EM patients and 18 age- and sex-matched normal controls at first. Secondly, the seed points of PAG subregions were defined, and the functional connectivity was computed. Last, analysis of covariance was performed with age and sex as covariates, and voxel-based correlation was also applied between the functional connectivity of PAG subregions and the clinical variables.

## Methods

### Subjects

Written informed consent was obtained from all participants according to the approval of the ethics committee of the local institutional review board. Eighteen EM patients (15 EM patients without aura and 3 EM patients with aura) were recruited from the International Headache Center, Department of Neurology, Chinese PLA General Hospital from 2014 to 2015. All the following inclusion criteria should be fulfilled: 1) EM is defined as migraine attack days being less than 15 days per month. The definition of migraine refers to 1.1 Migraine without aura and 1.2 Migraine with aura in (ICHD-III beta) [12]; 2) no migraine preventive medication used in the past 3 months; 3) absence of any chronic disorders, including hypertension, hypercholesterolemia, diabetes mellitus, cardiovascular diseases, cerebrovascular disorders, neoplastic diseases, infectious diseases, connective tissue diseases, other subtypes of headache, chronic pain other than headache, severe anxiety or depression preceding the onset of headache, psychiatric diseases, etc.; 4) absence of alcohol, nicotine, or other substance abuse. Eighteen NCs were recruited from the hospital’s staff and their relatives. Inclusion criteria were similar to those of patients, except for the first two items. NCs should never have had any primary headache disorders or other types of headache in the past year. The exclusion criteria were the following: cranium trauma, illness interfering with central nervous system function, psychotic disorder, and regular use of a psychoactive or hormone medication. The anxiety, depression, and cognitive function of all the participants were evaluated with the Hamilton Anxiety Scale (HAMA) [13], the Hamilton Depression Scale (HAMD) [14], and the Montreal Cognitive Assessment (MoCA) Beijing Version (http://www.mocatest.org). All the patients were given with the Visual Analogue Scale (VAS) and the Migraine Disability Assessment Scale (MIDAS). MRI scans were taken in the interictal stage at least 3 days after a migraine attack for EM patients. All the subjects were right-handed and underwent conventional MRI examination to exclude the subjects with cerebral infarction, malacia, or occupying lesions. Alcohol, nicotine, caffeine, and other substances were avoided for at least 12 h before MRI examination.

### MRI acquisition

Images were acquired on a GE 3.0 T MR system (DISCOVERY MR750, GE Healthcare, Milwaukee, WI, USA) and a conventional eight-channel quadrature head coil was used. All subjects were instructed to lie in a supine position, and formed padding was used to limit head movement. Conventional T2weighted image (TR = 5000 ms, TE = 113.4 ms, FOV = 24 cm × 24 cm, Matrix = 384 × 384) and T1-FLAIR (TR = 2040 ms, TE = 6.9 ms, FOV = 24 cm × 24 cm, Matrix = 384 × 256) were obtained first. Then, the rs-fMRI was performed, during which subjects were instructed to relax, keep their eyes closed, stay awake, remain still, and clear their heads of all thoughts. Functional images were obtained using a gradient echo-planar imaging (EPI) sequence (TR = 2000 ms, TE = 30 ms, flip angle = 90, slice thickness = 3 mm, slice gap = 1 mm, FOV = 24 cm × 24 cm, Matrix = 64 × 64), and 180 continuous EPI functional volumes were acquired axially over 6 min. Finally, a high resolution three-dimensional T1-weighted fast spoiled gradient recalled echo (3D T1-FSPGR) sequence was performed, which generated 360 contiguous axial slices [TR (repetition time) = 6.3 ms, TE (echo time) = 2.8 ms, flip angle = 15°, FOV (field of view) = 25.6 cm × 25.6 cm, Matrix = 256 × 256, slice thickness = 1 mm]. None of the subjects complained of any discomfort or fell asleep during scanning. No obvious structural damage was observed based on the conventional MR images.

### MR image processing

All MR structural and functional images were processed using Statistical Parametric Mapping 12 (SPM12) (http://www.fil.ion.ucl.ac.uk/spm) and the rs-fMRI data analysis toolkit (REST v1.8) [15] running under MATLAB 7.6 (The Mathworks, Natick, MA, USA). The data preprocessing was carried out as follows: (1) The first ten volumes of each functional time course was discarded to allow for T1 equilibrium and the participants to adapt; (2) Slice timing; (3) Head motion correction; (4) Spatial normalization. These steps were performed by SPM12. No subjects had head motion with more than 1.5 mm displacement in X, Y, and Z direction or 1.50 of any angular motion throughout the course of the scanning. The linear trend removal and temporal band-pass filtering (0.01–0.08 Hz) was performed by REST [15].

**Fig. 1:**
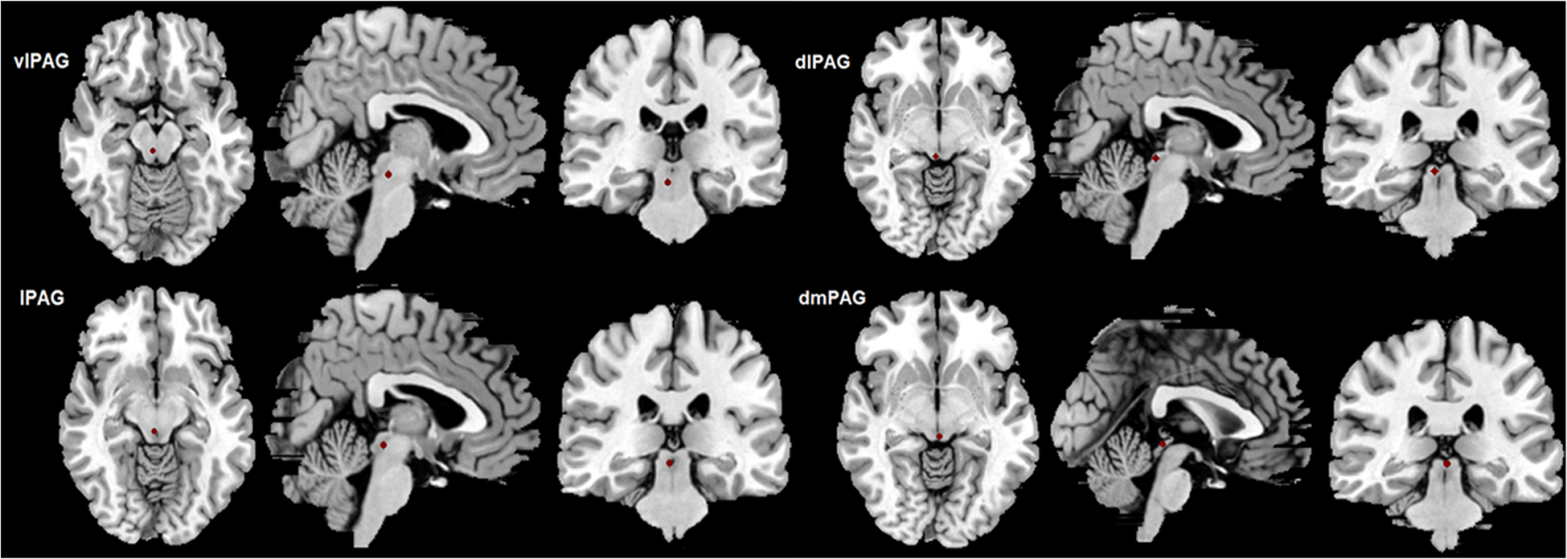
The seed points of the subregions of PAG. right vlPAG, MNI coordinate:[4–26 -14] with 2 mm radius; right lPAG, MNI coordinate: [3–28 -10] with 2 mm radius; right dlPAG, MNI coordinate: [3–30 -6] with 2 mm radius; dmPAG, MNI coordinate: [0–30 -6] with 2 mm radius

The functional connectivity analysis was performed as follows: (1) Spatial smooth (full width at half maximum (FWHM) = 6 mm) using SPM8; (2) The subregions of PAG were classified as seven seeds based on the previous studies [6, 10, 16–19]: 1) left ventrolateral PAG (l_vlPAG)(MNI coordinates: l_vlPAG = −4; −26; −14 with 2 mm radius); 2) right ventrolateral PAG(r_vlPAG) (MNI coordinates: r_vlPAG = 4; −26; −14 with 2 mm radius); 3) left lateral PAG(l_lPAG) (MNI coordinates: l_lPAG = −3; −28; −10 with 2 mm radius); 4) right lateral PAG (r_lPAG) (MNI coordinates: r_lPAG = 3; −28; −10 with 2 mm radius); 5) left dorsolateral PAG(l_dlPAG) (MNI coordinates: l_dlPAG = −3; −30; −6 with 2 mm radius); 6) right dorsolateral PAG (r_dlPAG) (MNI coordinates: l_dlPAG = 3; −30; −6 with 2 mm radius); 7) dorsomeidal PAG (dmPAG) (MNI coordinates: dmPAG = 0; −30; −6 with 2 mm radius) (Fig. 1); (3) Functional connectivity computation of the seven subregions of PAG were performed using REST (v1.8). The time courses of subregions of PAG were extracted, and Pearson’s correlations were used to calculate the functional connectivity between the extracted time courses and the averaged time courses of the whole brain in a voxel-wise manner. The white matter, cerebrospinal fluid (CSF), and the six head motion parameters were used as covariates. (4) The individual r-maps were normalized to Zmaps using Fisher’s Z-transformation.

### Statistical analysis

An independent sample *t*-test was applied to the comparison of the age, HAMA, HAMD and MoCA score between NC and EM group. Significant difference was set at a *P* value of < 0.05. The statistical analysis was performed using SPSS 19.0. Two-sample t-tes was performed to identify the regions with significant differences in connectivity to subregions of PAG, covarying for age and sex, and significance was set at a *P* value of <0.001 without correction. Voxel-based correlation analysis was performed using multiple regression model, and significance was set a *P* value of < 0.05 with FDR correction. The minimal number of contiguous voxels was set based on the expected voxels per cluster. The statistical analysis was performed by SPM 12 software.

## Results

### Demography and neuropsychological test

Eighteen EM patients (F/M = 14/4) and 18 NCs (F/M = 14/4) were enrolled. There was no significant difference for age between EM (33.39 ± 10.69 years old) and CM (39.11 ± 9.99 years old). There was a significant difference for HAMA between NC(9.67 ± 3.16) and EM (15.67 ± 9.85), HAMD between NC(10.89 ± 7.26) and EM(15.89 ± 2.89) and MoCA between NC (27.00 ± 2.46) and EM (29.17 ± 1.47) (*P* < 0.05)(Table 1).

**Table 1:** The clinical characteristics of normal controls and EM patients

	EM	NC
Num(F/M)	18(14/4)	18(14/4)
Age	33.39 ± 10.69	39.11 ± 9.99
HAMA	15.67 ± 9.85	9.67 ± 3.16
HAMD	15.89 ± 2.89	10.89 ± 7.26
MoCA	29.17 ± 1.47	27.00 ± 2.46
DD(yrs)	12.44 ± 8.07	NA
VAS	8.33 ± 1.50	NA
MIDAS	16 ± 17.94	NA

*EM* episodic migraine, *NC* normal control, *HAMA* hamilton anxiety scale, *HAMD* hamilton depression scale, *MoCA* montreal cognitive assessment, *DD* disease duration, *VAS* visual analogue scale, *MIDAS* migraine disability assessment questionnaire, *NA* not available

### Comparison of the functional connectivity of the subregions of PAG between NC and EM

Table 2 demonstrated that only right vlPAG, left vlPAG and left dlPAG presented decreased functional connectivity in EM compared with NC, and the other subregions (bilateral lPAGs, right dlPAG and dmPAG) showed no significant altered functional connectivity in EM compared with NC.

**Table 2:** The brain regions with altered functional connectivity of PAG over the whole brain between EM and NC

Group	BA	Anatomic region	MNI-space			Cluster size	*P*_uncorr_	Peak T value
			X	Y	Z			
Right_vlPAG								
NC > EM	BA6	Precentral_L	−21	−15	60	14	0.000	4.53
NC < EM		NA	NA	NA	NA	NA		NA
Left_vlPAG								
NC > EM	BA21	Temporal_Mid_R	48	−42	3	13	0.000	4.86
	BA6	Precentral_L	−36	−9	39	20	0.000	4.26
	BA6	Frontal_Sup_R	21	−9	66	24	0.000	3.9
	BA6	Supp_Motor_Area_R	3	12	63	12	0.000	4.16
	BA21	Temporal_Mid_L	−54	−51	9	12	0.000	4.16
	BA46	Frontal_Sup_R	27	48	12	15	0.000	4.14
	BA40	Parietal_Inf_L	−36	−39	42	17	0.000	4.09
	BA46	Frontal_Mid_L	−30	45	21	20	0.000	3.97
NC < EM		NA	NA	NA	NA	NA		NA
Right_lPAG								
NC > EM		NA	NA	NA	NA	NA		NA
NC < EM		NA	NA	NA	NA	NA		NA
Left_lPAG								
NC > EM		NA	NA	NA	NA	NA		NA
NC < EM		NA	NA	NA	NA	NA		NA
Right_dlPAG								
NC > EM		NA	NA	NA	NA	NA		NA
NC < EM		NA	NA	NA	NA	NA		NA
Left_dlPAG								
NC > EM	BA45	Frontal_Inf_Tri_R	48	27	9	12		5.14
	BA8	Frontal_Sup_Medial_R	6	27	57	14		5.13
NC < EM		NA	NA	NA	NA	NA		NA
dmPAG								
NC > EM		NA	NA	NA	NA	NA		NA
NC < EM		NA	NA	NA	NA	NA		NA

*NC* normal control, *EM* episodic migraine, *vlPAG* ventrolateral PAG, *lPAG* lateral PAG, *dlPAG* dorsolateral PAG, *dmPAG* dorsomedial PAG

**Fig. 2:**
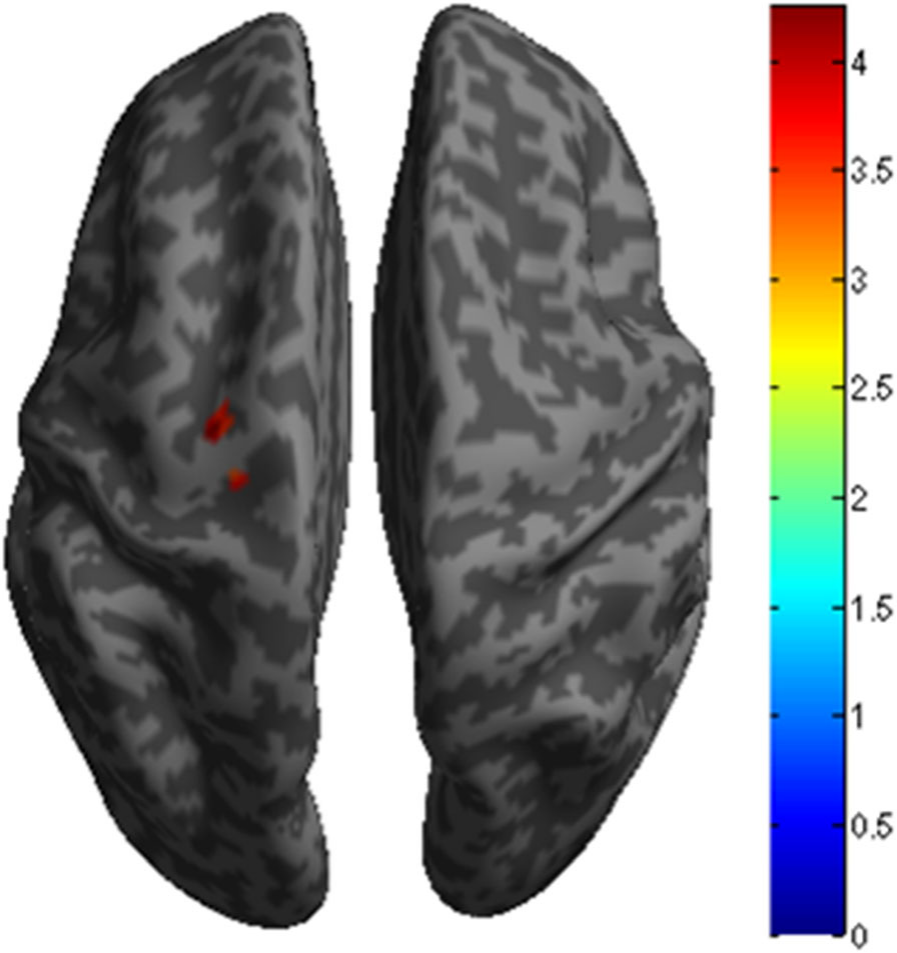
The decreased functional connectivity of right ventrolateral PAG in EM compared with NC

**Fig. 3:**
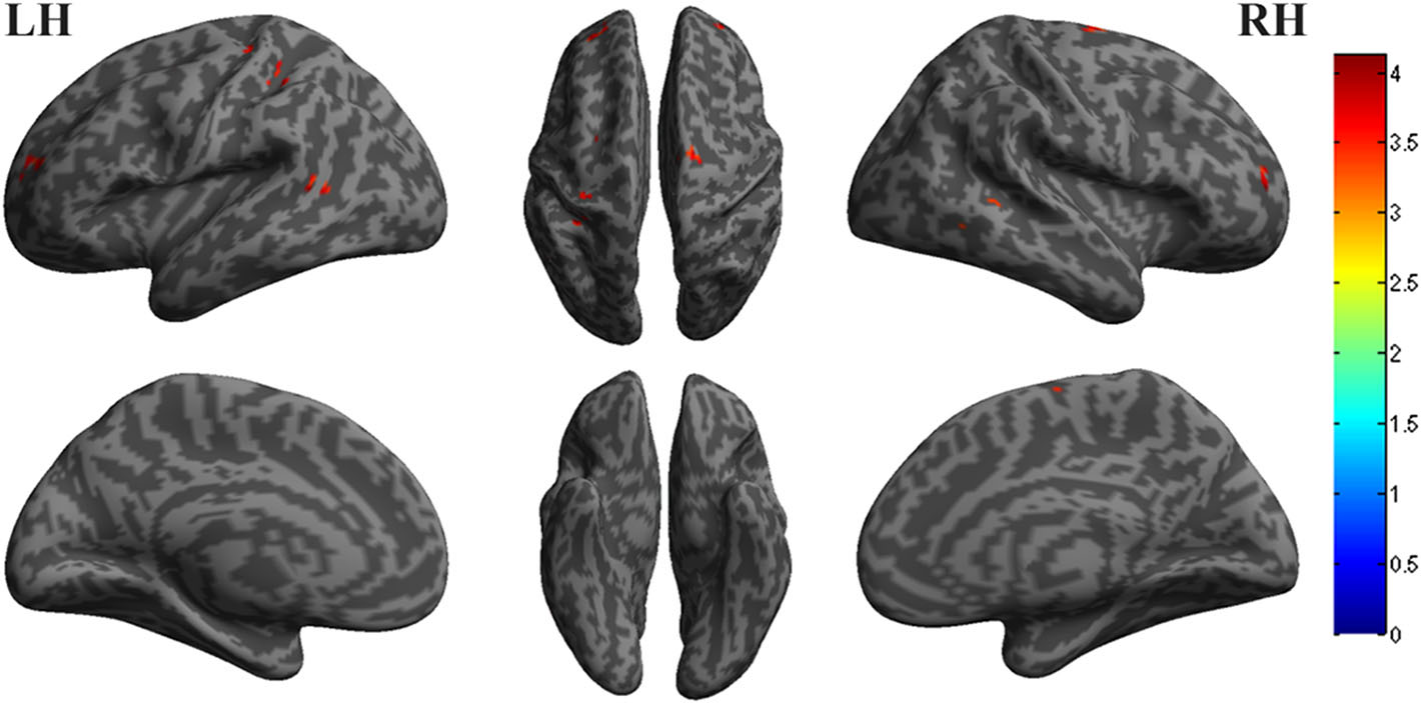
The decreased functional connectivity of left ventrolateral PAG in EM compared with NC

**Fig. 4:**
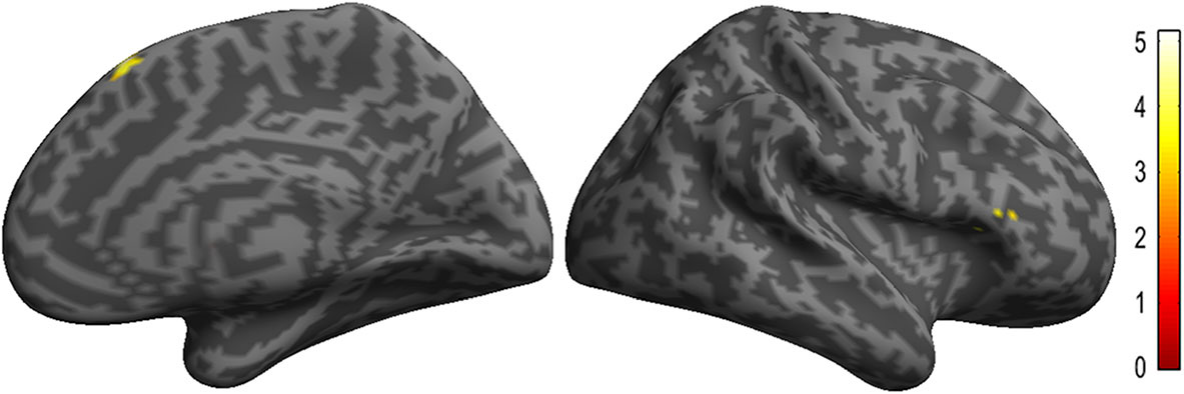
The decreased functional connectivity of left dorsolateral PAG in EM compared with NC

The decreased functional connectivity of right vlPAG located in left precentral gyrus (Fig. 2), and the decreased functional connectivity of left vlPAG located in left precentral gyrus, left middle frontal gyrus, left inferior parietal gyrus, bilateral middle temporal gyrus, right superior frontal gyrus and right supplementary motor area (Fig. 3). Figure 4 revealed that the decreased functional connectivity of left dlPAG located in the right parts triangularis of inferior frontal gyrus and medial superior frontal gyrus.

### Correlation analysis between the functional connectivity of the bilateral vlPAG and left dlPAG and the clinical variables

**Fig. 5:**
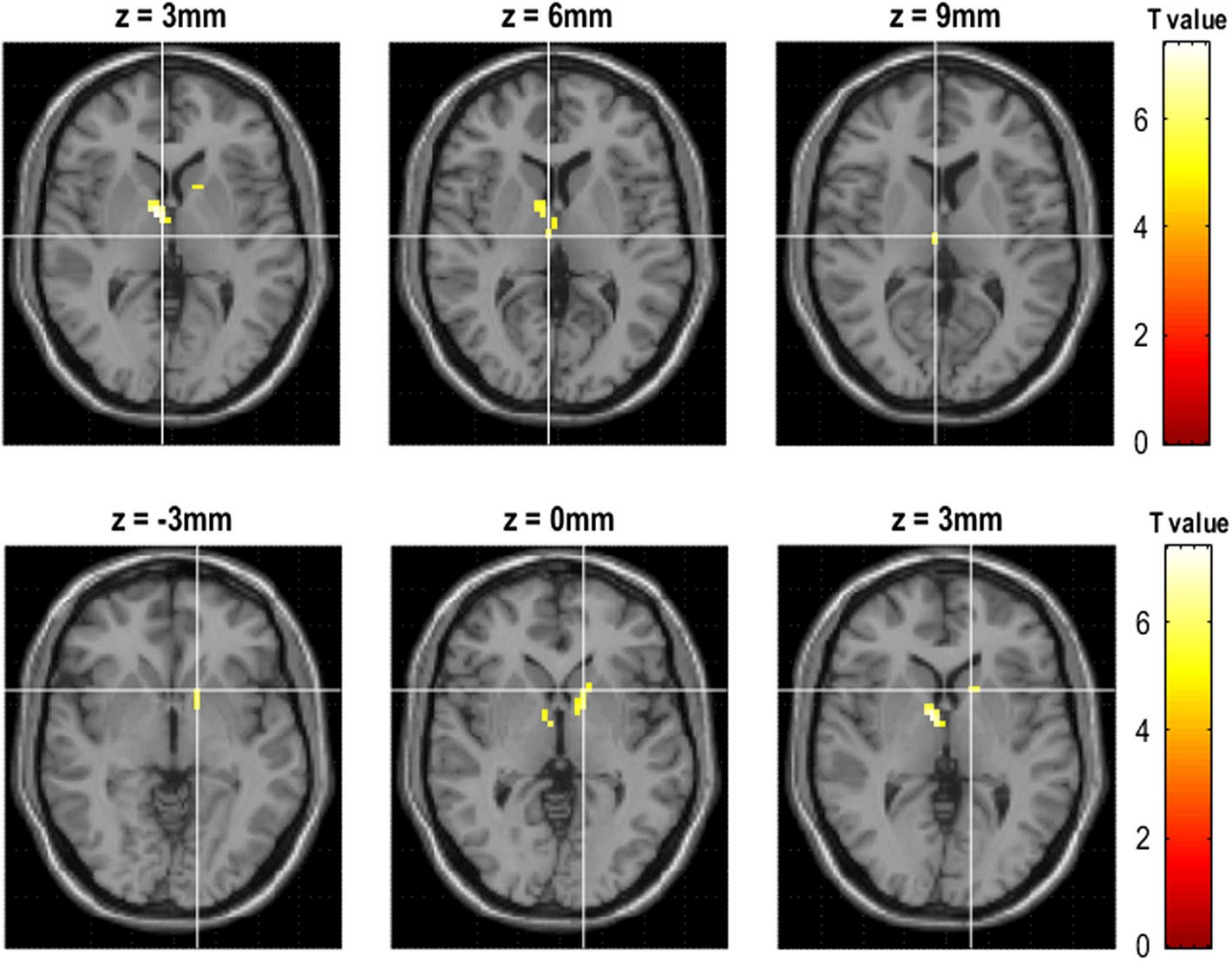
The brain regions with positive correlation between the functional connectivity of left ventrolateral PAG and disease duration in EM patients

**Fig. 6:**
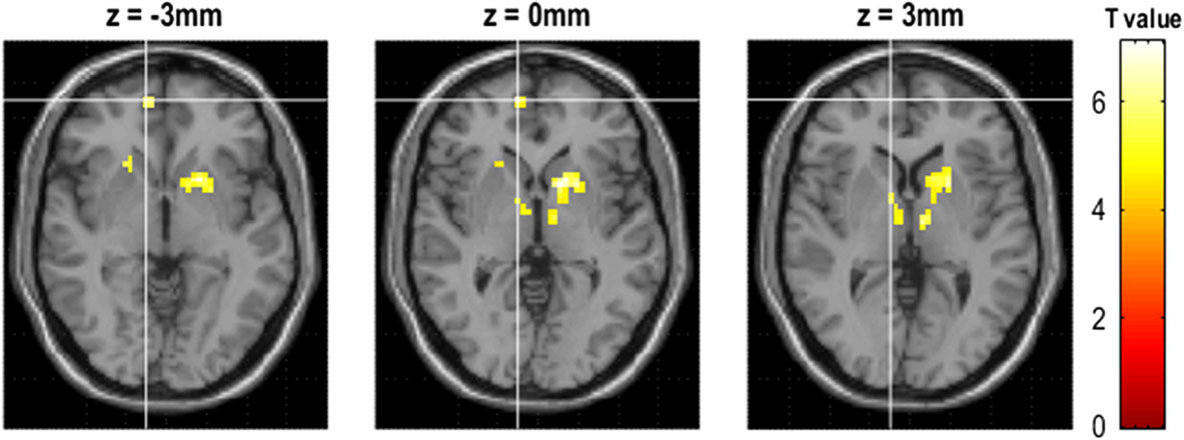
The brain regions with positive correlation between the functional connectivity of right ventrolateral PAG and disease duration in EM patients

Only disease duration was positively related with the functional connectivity of bilateral vlPAG, and the other clinical variables showed no significant correlation with the functional connectivity of vlPAG (Table 3). Figure 5 demonstrated that the brain regions with positive correlation located in bilateral thalamus and left pallidum between the functional connectivity of left vlPAG and disease duration in EM patients. The brain regions with positive correlation with disease duration located in bilateral thalamus, putamen, and right medial orbitofrontal gyrus for the functional connectivity of right vlPAG (Fig. 6). There was no significant correlation between the functional connectivity of left dlPAG and the clinical variables.

**Table 3:** The voxel-based correlation analysis between the functional connectivity of left ventrolateral PAG(vlPAG) and right vlPAG and the clinical variables

Group	Anatomic region	Cluster size	MNI-space			*P*_uncorr_	Peak T value
			X	Y	Z		
Left_vlPAG							
DD							
Positive							
	Thalamus_R	23	9	−3	3	0.000	7.39
			6	−15	6	0.000	5.38
	Pallidum_L	15	−12	3	0	0.000	5.8
	Thalamus_L		−12	−12	0	0.000	5.68
Right_vlPAG							
DD							
Positive							
	Putamen_L	104	−21	9	−9	0.000	8.75
	Frontal_Med_Orb_R	12	12	57	0	0.000	6.31
	Thalamus_L	14	−6	−12	3	0.000	6.06
	Putamen_R	14	24	21	−3	0.000	5.49
	Thalamus_R	15	6	−15	6	0.000	5.12

*vlPAG* ventrolateral PAG, *DD* disease duration, *Positive* positive correlation

## Discussion

This study demonstrated that bilateral vlPAG and left dlPAG may play a role in the descending pain modulatory circuit in EM patients, and the other PAG subregions did not show significant altered functional connectivity in EM. This altered functional connectivity pattern established a concept that PAG subregion analysis may be adapted to the investigation of the pain modulatory in EM while not a whole PAG region analysis. Based on the anatomical organization of PAG, different PAG subregions had different cytoarchitecture and neural connections, therefore, different PAG subregions presented different physiological function [20, 21], which may be used to explain the altered functional connectivity of some selected PAG subregions.

In this study, bilateral vlPAG dysfunction were involved with the disrupted functional connectivity in EM patients, especially presented the left predominance. It was known that PAG contains neural circuits that participate in descending antinociception [22], and vlPAG release the antinociceptive peptides Met-enkephalin and neurotensin, which were released when nociception occurred and would result in greater sensitivity of PAG enkephalinergic neurons and greater excitability of the descending PAG output neurons that are responsible for antinociception [23]. The output of vlPAG neurons could also be influenced by GABA recptors, and would disrupt the descending pain control system to spinal dorsal horn in a rat experiments [24]. Furthermore, PAG regions receive the projection from spinothalamic tract via the spinomesencephalic tract to transmit the ascending pain and temperature information [25]. Therefore, vlPAG dysfunction may be considered as an important targeted impaired PAG subregion in EM patients.

In this study, right vlPAG only presented the decreased functional connectivity with left precentral gyrus (BA6, premotor cortex), and left vlPAG showed the decreased functional connectivity with left precentral gyrus, right superior frontal gyrus (BA6, premotor cortex, PMC) and right supplementary motor area (SMA). The decreased functional connectivity between motor cortex and PAG was also demonstrated in fibromyalgia patients [26], which was also demonstrated in this study and it suggested that vlPAG dysfunction manifested as decreased contralateral functional output from premotor and SMA, and the neuromechanism should further be elucidated.

Left vlPAG presented the decreased functional connectivity with bilateral mid-dorsolateral prefrontal cortex (dlPFC) (BA46) and left dlPAG presented the decreased functional connectivity with right posterior dlPFC (BA8) based on the functional parcellation of PFC. dlPFC mainly lies in the middle frontal gyrus, and it is connected to multiple brain regions, thalamus, basal ganglia and hippocampus, and its function included working memory [27], cognitive flexibility [28], planning [29] and emotion [30]. Although previous document confirmed that PFC dysfunction may be impaired in migraine [7, 31], the precise PFC functional location was not involved in these studies, and a recent study demonstrated that migraine without aura showed decreased functional connectivity between PAG and medial PFC (mPFC). In this study, mid-dlPFC and posterior dlPFC presented decreased functional connectivity with left vlPAG and left dlPAG in EM patients, which might be associated with higher HAMA and HAMD scores in EM patients since the dlPFC participated the emotion-nmodulated performance and activity [30]. Therefore, this study suggested that dlPFC might be the targeted impaired brain region in the disrupted vlPAG dysfunction network.

Also, the left dlPAG showed the decreased functional connectivity with right ventrolateral prefrontal cortex (vlPFC) (BA45) in EM patients (Fig. 4). vlPFC was located in the inferior frontal gyrus, and including anterior (BA47), middle (BA45) and posterior (BA44) PFC, which presented different function [32–34]. It was known that right and left vlPFC showed the different specific functions [35], and right vlPFC was thought be play a key role in the motor control [36], and its involvement was not still reported in migraine, and it may be associated with the study methods. In a previous study [37], right anterior vlPFC (BA47) was involved in the integration of emotional processing using a parametric mediation analysis of fMRI. However, right middle (BA45) vlPFC presented decreased functional connectivity with left dlPAG region in the current study. Although it was not consistent with the previous document, it might speculate that middle vlPFC participate in the emotional processing in EM patients since the HAMA and HAMD scores were higher in migraineurs.

Based on two-streams hypothesis [38, 39], dlPFC was the endpoint of the dorsal stream, and vlPFC was the endpoint of the ventral stream. Herein, it could recognize that the endpoints of dorsal and ventral stream were disrupted for the functional connectivity with vlPAG and dlPAG in EM patients, and which may be associated with the visual processing in migraine. In this study, bilateral middle temporal gyrus (BA21) presented decreased functional connectivity with left vlPAG, and BA21 region mainly take part in the processing of visual information, which indicated that disrupted visual information processing may occur in EM patients, and may be associated ventral and dorsal stream.

Voxel-based correlation analysis demonstrated the positive correlation of the diseased duration with the altered functional connectivity of vlPAG, while clinical variables such as VAS, onset frequence, and neuropsychological scale scores were not related with the PAG functional connectivity. These findings indicated that vlPAG dysfunction may be associated with the disease duration, and disease duration may indirectly be used to predict the severity of vlPAG dysfunction in EM patients. A previous study demonstrated that basal ganglia played a significant role in the pathophysiology of the episodic migraine [40], and it also revealed that the disrupted connection with the subcortical gray matter structures (bilateral thalamus and putamen) may be the primary impaired neuromechanism for the descending pain modulation for vlPAG in EM patient.

## Conclusions

In conclusion, disrupted functional connectivity of bilateral vlPAG and left dlPAG presented in bilateral dlPFC, right vlPFC, blilateral PMC and right SMA, bilateral middle temporal gyri in EM patients, and the disease duration was positively related to the functional connectivity of bilateral vlPAG on the bilateral thalamus and putamen, left pallidum and right medial orbitofrontal gyrus in EM patients. These findings suggested that functional evaluation of PAG subregion may be contributed to the diagnosis and understanding of EM pathogenesis.

## Abbreviations

dlPAG: Dorsolateral PAG
dmPAG: Dorsomedial PAG
EM: Episodic migraine
lPAG: Lateral PAG
NC: Normal controls
PAG: Periaqueductal gray
PFC: Prefrontal cortex
PMA: Primary motor area
SMA: Supplementary motor area
vlPAG: Ventrolateral PAG
